# Nonlinear Association Between Serum 25‐Hydroxyvitamin D and Cardiac Autonomic Dysfunction in Diabetic Foot: A Threshold Effect on Heart Rate Variability

**DOI:** 10.1111/1753-0407.70109

**Published:** 2025-06-10

**Authors:** Mingxin Bai, Donge Yan, Ruixue Feng, Xingwu Ran, Dawei Chen, Chun Wang, Lihong Chen, Shuang Lin, Sen He, Yan Liu, Murong Wu, Zhiyi Lei, Yun Gao

**Affiliations:** ^1^ Diabetic Foot Care Center, Department of Endocrinology and Metabolism West China Hospital, Sichuan University Chengdu Sichuan People's Republic of China; ^2^ Department of Cardiology West China Hospital, Sichuan University Chengdu Sichuan People's Republic of China; ^3^ Department of Endocrinology Chengdu Eighth People's Hospital Chengdu Sichuan People's Republic of China; ^4^ West China Medical School, Sichuan University Chengdu Sichuan People's Republic of China

**Keywords:** 25‐hydroxyvitamin D, cardiac autonomic dysfunction, diabetic foot, heart rate variability

## Abstract

**Background:**

Previous studies have shown that vitamin D deficiency was associated with both cardiac autonomic dysfunction and the development of diabetic foot (DF). However, the impact of vitamin D levels on heart rate variability (HRV) in individuals with DF, a high‐risk group, remains unclear. We explored the association between vitamin D status and HRV in individuals with DF.

**Methods:**

A total of 458 individuals with DF were assessed for vitamin D levels by 25‐hydroxyvitamin D (25(OH)D) and evaluated for cardiovascular autonomic function using both time and frequency domains of the HRV measures. Multivariate regression analysis and restricted cubic spline regression were employed to examine the associations.

**Results:**

Vitamin D levels were positively associated with HRV indices in people with DF, including standard deviation of the normal sinus interval (SDNN), standard deviation of the 5‐min average RR intervals (SDANN), low‐frequency power (LF), high‐frequency power (HF), and the LF/HF ratio (all *p* < 0.05). The associations between serum 25(OH)D and cardiac autonomic dysfunction were nonlinear. When 25(OH)D levels were < 50 nmol/L, the odds ratio (OR) for predicted cardiac autonomic dysfunction per SD increase in 25(OH)D was 0.56 (95% CI, 0.44–0.72). However, no significant effect was observed when 25(OH)D levels exceeded 50 nmol/L.

**Conclusions:**

This study demonstrates that lower 25(OH)D levels are associated with reduced HRV in individuals with DF. Specifically, when 25(OH)D levels fall below 50 nmol/L, the risk of cardiac autonomic dysfunction in people with DF significantly increases.

## Graphical abstract

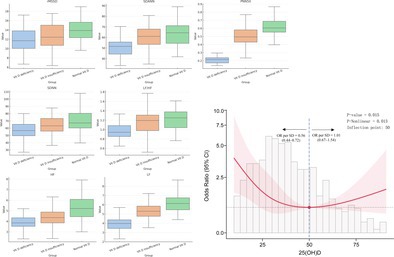

**Summary**

- Lower 25(OH)D levels were associated with impaired HRV in individuals with DF, a high‐risk group.
- A nonlinear relationship showed that when 25(OH)D levels < 50 nmol/L, the risk of cardiac autonomic dysfunction significantly increases in individuals with DF.

## 1 Introduction

Vitamin D deficiency has emerged as a global health crisis affecting over 1 billion individuals worldwide, with particularly high prevalence among people with chronic metabolic disorders such as diabetes mellitus [1]. Beyond its classical role in calcium‐phosphorus homeostasis, accumulating evidence reveals vitamin D as a pleiotropic hormone with critical cardiovascular and neuroprotective functions [2, 3]. Increasing evidence suggests that vitamin D is a strong risk marker for cardiovascular diseases and for cardiovascular risk factors such as diabetes mellitus [4, 5, 6]. Moreover, some studies have reported impaired cardiac autonomic function in individuals with vitamin D deficiency, highlighting its essential role in maintaining cardiac autonomic homeostasis [7, 8, 9, 10, 11]. These findings reinforce the hypothesis that low vitamin D levels contribute to increased cardiovascular risk by disrupting the regulatory function of the cardiac autonomic nervous system.

Cardiovascular autonomic neuropathy (CAN), characterized by impaired autonomic regulation of the cardiovascular system, is also a common complication of diabetes. It is strongly associated with an increased risk of cardiac arrhythmias, sudden death, and possibly related to silent myocardial ischemia [12]. The presence of CAN may increase the risk for cardiovascular‐related mortality, as well. Heart rate variability (HRV) is considered a reliable and sensitive tool for quantifying the sympathetic and parasympathetic components of the autonomic nervous system [13]. Reduced HRV has been associated with an increased risk of cardiovascular events, all‐cause mortality, and the progression of diabetic complications such as diabetic neuropathy and foot ulcers [14].

Diabetic foot (DF), the most severe complication of diabetes, often leads to significant morbidity and mortality, affecting patients' quality of life [15]. Notably, prior studies have reported individuals with DF tend to have lower levels of vitamin D, and this deficiency is associated with poorer outcomes, including impaired wound healing and increased risk of infection [16, 17]. Additionally, it has been reported that the prevalence of CAN is three to five times higher in individuals with DF compared to those without foot complications [18, 19]. Similarly, our previous research demonstrated significantly lower HRV in individuals with DF, further supporting the association between DF and autonomic dysfunction [20]. Thus, people with DF appear to be at high risk for both cardiac autonomic dysfunction and vitamin D deficiency. However, limited studies have explored the relationship between vitamin D levels and cardiac autonomic function specifically in this population. Therefore, our study aims to investigate the association between vitamin D status and HRV in individuals with DF, providing insights into the potential role of vitamin D in modulating cardiac autonomic function and its clinical implications for managing cardiovascular risk in this high‐risk group.

## 2 Methods

### 2.1 Study Population

**FIGURE 1:**
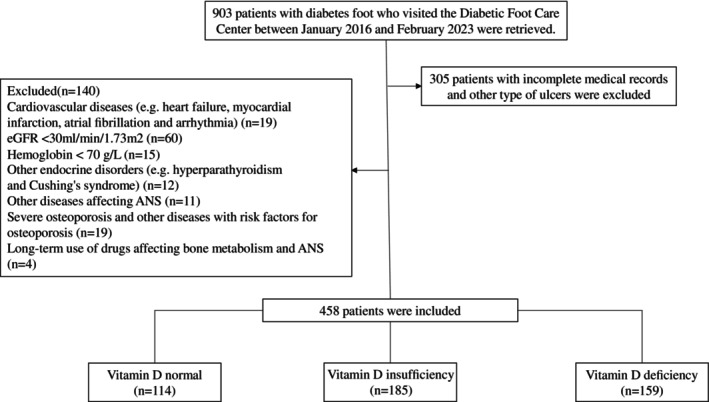
Flow chart of the study. ANS, autonomic nervous system; eGFR, estimated glomerular filtration rate.

We retrospectively reviewed the medical records of 903 individuals with DF who visited the Diabetic Foot Care Center of West China Hospital, Sichuan University between January 2016 and February 2023, excluding 305 individuals with incomplete records or other types of ulcers. Patients aged over 18 years who met the diagnostic criteria for DF as defined by the 2019 International Working Group on the Diabetic Foot (IWGDF) expert consensus were eligible for inclusion in this study [21]. Exclusion criteria are as follows: severe cardiovascular diseases (e.g., congenital heart disease, acute myocardial infarction, cardiac arrhythmias, acute heart failure); a history of cardiac surgery within the past 6 months; estimated glomerular filtration rate (eGFR) < 30 mL/min/1.73 m^2^; uncontrolled thyroid or parathyroid dysfunction or other endocrine disorders; hemoglobin < 70 g/L; conditions affecting autonomic function, such as active rheumatic or autoimmune disease, hematologic disease, infection, central nervous system, or psychiatric disease; severe osteoporosis or other conditions affecting vitamin D metabolism, such as malignancy, hepatic insufficiency, or inflammatory bowel disease; chronic use of medications that affect autonomic function or vitamin D levels, including tricyclic antidepressants, β‐blockers, amiodarone, or vitamin D supplements; current pregnancy or lactation. After applying these criteria, 458 individuals with DF were ultimately included in the study (Figure 1). The study was reviewed and approved by the Institutional Ethics Committee of the West China Hospital and was registered in the Clinical Trial Registry (registration number: CHiCTR2300076628).

### 2.2 Assessment of Serum 25‐Hydroxyvitamin D (25(OH)D)

25(OH)D is the main circulating form of vitamin D. To assess vitamin D status, serum 25(OH)D levels were measured in blood samples obtained from the antecubital vein following a 12‐h overnight fast during routine health examinations. The total serum concentration of 25(OH)D was determined using an electrochemiluminescence immunoassay (Roche Cobas e601 analyzer). According to the recommendations from the Institute of Medicine (IOM) [22], we defined normal serum levels of 25(OH)D as ≥ 50 nmol/L, vitamin D insufficiency as 30 nmol/L ≤ 25(OH)D < 50 nmol/L, and vitamin D deficiency as 25(OH)D < 30 nmol/L.

### 2.3 Assessment of HRV

To evaluate HRV, we conducted 24‐h Holter electrocardiogram (ECG) monitoring. Prior to the assessment, participants were instructed to refrain from consuming alcohol and caffeine (i.e., tea or coffee) to enhance data reliability. The Holter monitor was placed between 8:00 and 10:00 a.m., and during the monitoring period, subjects carried out their normal daily activities. HRV indices were automatically computed using a Holter Analysis Workstation (GE Medical Systems Information Technologies Inc., El Paso, TX, USA), which adheres to the assessment standards and physiological interpretations established by the Task Force of the European Society of Cardiology and the North American Society of Pacing and Electrophysiology [23]. As the time‐domain indices, the standard deviation of the normal sinus interval (SDNN), the standard deviation of the 5‐min average RR intervals (SDANN), the root mean square of successive RR interval differences (rMSSD), and the percentage of normal adjacent RR interval difference > 50 ms (PNN50) were examined. As the frequency‐domain indices, the low‐frequency power (LF, frequency strength of 0–0.04 Hz), the high‐frequency power (HF, frequency strength of 0.15–0.4 Hz), and the LF/HF ratio were reported. An SDNN value < 50 ms was used to define cardiac autonomic dysfunction, consistent with previous studies based on 24‐h ECG data, and has been associated with increased cardiovascular risk [24].

### 2.4 Assessment of Covariates

Blood samples were taken after overnight fasting. Serum biochemical indices, such as glycated hemoglobin (HbA1c), total cholesterol (TC), triglycerides (TG), high‐density lipoprotein cholesterol (HDL‐C), low‐density lipoprotein cholesterol (LDL‐C), eGFR, alanine transferase (ALT), and aspartate transferase (AST) were measured by standard laboratory methods. Hypertension was defined as follows: systolic blood pressure (SBP) ≥ 140 mmHg or diastolic blood pressure (DBP) ≥ 90 mmHg, or use of antihypertension medication, or self‐reported physician diagnosis of hypertension [25]. The diagnosis of diabetic peripheral neuropathy (DPN) was based on clinical assessment, physical examination, and neurophysiologic tests [26]. Peripheral arterial disease (PAD) was defined by an ankle‐brachial index (ABI) < 0.9, confirmed imaging examination, or history of revascularization therapy [27, 28]. The diagnosis of diabetic retinopathy (DR) was documented by an ophthalmologist, and diabetic nephropathy (DN) required evidence of micro‐ or macroalbuminuria. Diagnoses of coronary artery disease (CAD) and cerebrovascular disease (CVD) were abstracted from the clinical notes of the patient.

### 2.5 Statistical Analysis

Categorical variables were expressed as percentages, and continuous variables were described as mean ± standard deviation (SD) for normally distributed data or median (interquartile range) for skewed data. Group comparisons for continuous variables were performed using Welch's *t*‐test or ANOVA for normally distributed data and the Wilcoxon rank‐sum test or Kruskal–Wallis test for non‐normally distributed data. Categorical data were compared using the Fisher exact test or Chi‐squared test. *p* for trend was evaluated using linear or logistic regression, incorporating 25(OH)D either as a continuous variable or as an ordinal variable categorized into deficiency, insufficiency, and sufficiency. To improve normality for regression analyses, the HRV variables SDNN, SDANN, rMSSD, LF, and HF were natural log‐transformed, while PNN50 was square root‐transformed. Logistic regression models were used to assess the association between 25(OH)D and cardiac autonomic dysfunction, adjusting for sex, age, diabetic duration, smoking history, body mass index (BMI), mean blood pressure (MBP), HbA1c, TC, TG, eGFR, and comorbidities such as DN, DR, DPN, PAD, CVD, CAD, and hypertension (HBP). Additionally, the nonlinear association between 25(OH)D and cardiac autonomic dysfunction was modeled by restricted cubic spline (RCS) function using four knots (5th, 35th, 65th, 95th percentiles). All statistical analyses were performed using R software (version 4.2.2) and SAS 9.4 software. A two‐sided *p*‐value of < 0.05 was considered statistically significant.

## 3 Results

### 3.1 Characteristics of Study Population

A total of 458 individuals (302 males and 156 females) with an average age of 64.8 ± 12.0 years participated in this study. The mean 25(OH)D level was 39.61 ± 19.76 nmol/L. Of the participants, 34.7% had vitamin D deficiency (< 30 nmol/L), and 40.4% had vitamin D insufficiency (30–50 nmol/L). Table 1 shows the individuals' clinical characteristics according to the vitamin D status. Individuals in the vitamin D deficiency group had lower levels of TC, LDL‐C, HDL‐C, and eGFR, and higher levels of blood pressure and HbA1c. In addition, individuals in the vitamin D deficiency or insufficiency groups more often had a history of DR, DN, DPN, and HBP compared to those with normal vitamin D levels.

**TABLE 1:** Baseline demographic and clinical characteristics.

	Vitamin D deficiency (*n* = 159)	Vitamin D insufficiency (*n* = 185)	Vitamin D normal (*n* = 114)	*p*
Sex, men (%)	103 (64.8)	121 (65.4)	78 (68.4)	0.806
Age (yr)	65.0 (54.0–73.5)	64.0 (55.0–74.0)	67.5 (61.0–73.8)	0.115
Diabetic duration (yr)	12 (7–16)	11 (5–16)	12 (7–19)	0.264
Smoking, yes (%)	81 (50.9)	85 (45.9)	55 (48.2)	0.652
BMI (kg/m^2^)	23.40 (21.63–25.40)	23.70 (22.00–25.40)	23.35 (21.60–25.41)	0.723
SBP (mmHg)	144 ± 22	139 ± 22	139 ± 21	0.080
DBP (mmHg)	82 (73–90)	79 (72–87)	79 (71–88)	0.115
MBP (mmHg)	103 ± 14	100 ± 13	99 ± 13	0.065
HbA1c (%)	8.2 (7.1–10.2)	7.8 (6.8–9.4)	7.8 (7.0–9.2)	0.188
TC (mmol/L)	3.39 (2.79–4.39)	3.96 (3.16–4.81)	3.93 (3.13–4.51)	0.010
TG (mmol/L)	1.25 (1.00–1.75)	1.38 (0.97–1.85)	1.35 (1.07–1.78)	0.397
HDL‐C (mmol/L)	0.93 (0.80–1.21)	1.05 (0.89–1.29)	1.03 (0.87–1.30)	0.007
LDL‐C (mmol/L)	1.96 (1.42–2.75)	2.29 (1.69–3.05)	2.30 (1.59–2.90)	0.005
eGFR (mL/min/1.73 m^2^)	77.61 (53.70–95.68)	84.06 (61.10–99.83)	84.33 (62.99–95.55)	0.177
ALT (U/L)	17.0 (12.0–23.0)	18.0 (12.0–26.0)	16.5 (11.0–28.8)	0.791
AST (U/L)	18.0 (14.5–25.0)	18.0 (14.0–25.0)	18.0 (14.0–25.0)	0.970
LVEF (%)	66.0 (62.0–70.0)	68.0 (63.0–71.0)	66.0 (63.0–70.0)	0.160
DR (%)	85 (53.5)	86 (46.5)	52 (45.6)	0.326
DN (%)	68 (42.8)	59 (31.9)	31 (27.2)	0.018
DPN (%)	156 (98.1)	179 (96.8)	109 (95.6)	0.483
PAD (%)	67 (42.1)	67 (36.2)	55 (48.2)	0.117
CVD (%)	12 (7.5)	15 (8.1)	10 (8.8)	0.935
CAD (%)	32 (20.1)	26 (14.1)	29 (25.4)	0.046
HBP (%)	114 (71.7)	93 (50.3)	71 (62.3)	< 0.001

*Note:* Data are mean ± SD, median (IQR), or *n* (percentage).

Abbreviations: ALT: alanine transferase; AST: aspartate transferase; BMI: body mass index; CAD: coronary artery disease; CVD: cerebrovascular disease; DBP: diastolic blood pressure; DN: diabetic nephropathy; DPN: diabetic peripheral neuropathy; DR: diabetic retinopathy; eGFR: estimated glomerular filtration rate; HbA1c: glycated hemoglobin; HBP: hypertension; HDL‐C: high‐density lipoprotein cholesterol; LDL‐C: low‐density lipoprotein cholesterol; LVEF: left ventricular ejection fraction; MBP: mean blood pressure; PAD: peripheral arterial disease; SBP: systolic blood pressure; TC: total cholesterol; TG: triglycerides.

### 3.2 Vitamin D Status and HRV Indices

Table 2 shows HRV indices in individuals with DF at different vitamin D statuses. Compared to the normal vitamin D group, both time‐domain and frequency‐domain HRV indices were significantly reduced in the vitamin D insufficiency/deficiency groups. SDNN, SDANN, PNN50, HF, LF, and the LF/HF ratio were significantly lower in the vitamin D deficiency group than in the other two groups (*p* < 0.05). RMSSD was also slightly lower in the vitamin D insufficiency/deficiency groups, but the difference was not statistically significant (*p* = 0.08).

**TABLE 2:** Indices of heart rate variability in individuals with diabetic foot at different 25(OH)D status.

	Vitamin D deficiency (*n* = 159)	Vitamin D insufficiency (*n* = 185)	Vitamin D normal (*n* = 114)	*p*
HR (bpm)	82.4 ± 10.45	80.4 ± 10.21	78.6 ± 11.71	0.012
SDNN (ms)	55.0 (43.0–74.5)	66.0 (53.0–89.0)	72.0 (49.0–93.8)	< 0.001
SDANN (ms)	51.0 (39.0–66.5)	60.0 (48.0–81.0)	65.0 (43.3–86.0)	< 0.001
PNN50 (%)	0.2 (0.0–1.6)	0.5 (0.0–2.4)	0.6 (0.1–3.1)	0.031
rMSSD (ms)	12.0 (9.0–18.5)	13.0 (10.0–19.0)	15.0 (10.0–22.0)	0.080
HF (ms^2^)	4.0 (2.9–6.4)	4.5 (3.4–6.9)	5.1 (3.2–7.6)	0.009
LF (ms^2^)	3.9 (2.3–7.0)	5.4 (3.3–8.9)	5.9 (3.3–10.2)	< 0.001
LF/HF	1.0 (0.7–1.3)	1.2 (0.9–1.5)	1.2 (0.9–1.6)	< 0.001

*Note:* Data are mean (SD), median (IQR), or *n* (percentage).

Abbreviations: 25(OH)D: 25‐hydroxyvitamin D; HF: high‐frequency power; HR: heart rate; LF: low‐frequency power; LF/HF: rate of low‐frequency power between high‐frequency power; PNN50: the percentage of normal adjacent RR interval difference > 50 ms; rMSSD: the root mean square of successive RR interval differences; SDNN: the standard deviation of normal sinus interval; SDANN: the standard deviation of the 5‐min average RR intervals.

Further adjustments for sex, age, diabetic duration, smoking history, BMI, MBP, HbA1c, TC, TG, eGFR, and comorbidities such as DN, DR, DPN, PAD, CVD, CAD, and HBP showed that higher 25(OH)D levels remained significantly associated with higher HRV indices, as indicated by higher SDNN, SDANN, HF, LF, and the LF/HF ratio (*p* < 0.05). However, no significant associations were found between 25(OH)D levels and PNN50 or rMSSD. In terms of vitamin D status, compared to the normal vitamin D group, no significant differences in HRV indices were observed in the vitamin D insufficiency group, while the vitamin D deficiency group had significantly lower *β* values for SDNN (*β* = −0.067, 95% CI: −0.109, −0.024), SDANN (*β* = −0.065, 95% CI: −0.109, −0.022), LF (*β* = −0.107, 95% CI: −0.178, −0.037), and the LF/HF ratio (*β* = −0.059, 95% CI: −0.101, −0.016), suggesting poorer cardiac autonomic function (*p* for trend < 0.05) (Table 3). Additionally, we also found that reduced SDNN in people with DF is significantly associated with higher HbA1c levels, lower BMI, and the presence of DR, DN, and PAD (*p* < 0.05) (Table S1).

**TABLE 3:** Associations between 25(OH)D and indices of heart rate variability in individuals with diabetic foot.

	*β* (95% CI)						
	lg SDNN	lg SDANN	sqrt PNN50	lg rMSSD	lg HF	lg LF	lg(LF/HF)
25(OH)D	**0.001 (0.001 to 0.002)**	**0.001 (0.001 to 0.002)**	0.003 (−0.002 to 0.009)	0.001 (0.000 to 0.002)	**0.001 (0.000 to 0.002)**	**0.003 (0.001 to 0.004)**	**0.001 (0.000 to 0.002)**
*p*	< 0.001	0.001	0.205	0.130	0.022	< 0.001	0.004
Vitamin D normal	Ref	Ref	Ref	Ref	Ref	Ref	Ref
Vitamin D insufficiency	−0.007 (−0.048 to 0.033)	−0.007 (−0.049 to 0.035)	0.019 (−0.245 to 0.283)	0.001 (−0.046 to 0.049)	−0.001 (−0.054 to 0.052)	−0.011 (−0.078 to 0.057)	−0.005 (−0.046 to 0.035)
Vitamin D deficiency	**−0.067 (−0.109 to −0.024)**	**−0.065 (−0.109 to −0.022)**	−0.022 (−0.299 to 0.255)	−0.008 (−0.057 to 0.042)	−0.047 (−0.103 to 0.008)	**−0.107 (−0.178 to −0.037)**	**−0.059 (−0.101 to −0.016)**
*p* for trend	0.001	0.002	0.859	0.739	0.080	0.002	0.005

*Note:* Bold font indicates statistically significant differences. *β* and *p* values were generated using multivariate linear regression models with adjustment for imbalance variables, including sex, age, diabetic duration, smoking history, BMI, MBP, HbA1c, TC, TG, eGFR, DN, DR, DPN, PAD, CVD, CAD, and HBP.

Abbreviations: 25(OH)D: 25‐hydroxyvitamin D; BMI: body mass index; CAD: coronary artery disease; CVD: cerebrovascular disease; DN: diabetic nephropathy; DPN: diabetic peripheral neuropathy; DR: diabetic retinopathy; HbA1c: glycated hemoglobin; HBP: hypertension; HF: high‐frequency power; LF: low‐frequency power; LF/HF: rate of low‐frequency power between high‐frequency power; MBP: mean blood pressure; PAD: peripheral arterial disease; PNN50: the percentage of normal adjacent RR interval difference > 50 ms; rMSSD: the root mean square of successive RR interval differences; SDANN: the standard deviation of the 5‐min average RR intervals; SDNN: the standard deviation of normal sinus interval; TC: total cholesterol; TG: triglycerides.

Cardiac autonomic dysfunction, defined by an SDNN < 50 ms, was more prevalent in the vitamin D deficiency group than in the other groups (58/159 [36.5] vs. 36/185 [19.5] vs. 30/114 [26.3]). However, after adjustments for potential confounders in the multivariate logistic regression analysis, no significant association was observed between 25(OH)D levels and cardiac autonomic dysfunction in individuals with DF (Table 4).

**TABLE 4:** Logistic regression analysis of 25(OH)D and cardiac autonomic dysfunction in individuals with diabetic foot.

	OR (95% CI)	*p*
25(OH)D	0.992 (0.980–1.004)	0.174
Vitamin D normal	Ref	
Vitamin D insufficiency	0.668 (0.367–1.216)	0.187
Vitamin D deficiency	1.312 (0.731–2.355)	0.362
*p* for trend		0.230

*Note:* Multivariate logistic regression models with adjustment for imbalance variables, including sex, age, diabetic duration, smoking history, BMI, MBP, HbA1c, TC, TG, eGFR, DN, DR, DPN, PAD, CVD, CAD, and HBP.

Abbreviations: 25(OH)D: 25‐hydroxyvitamin D; OR: odds ratio; BMI: body mass index; CAD: coronary artery disease; CI: confidence interval; CVD: cerebrovascular disease; DN: diabetic nephropathy; DPN: diabetic peripheral neuropathy; DR: diabetic retinopathy; HbA1c: glycated hemoglobin; HBP: hypertension; MBP: mean blood pressure; PAD: peripheral arterial disease; TC: total cholesterol; TG: triglycerides.

**FIGURE 2:**
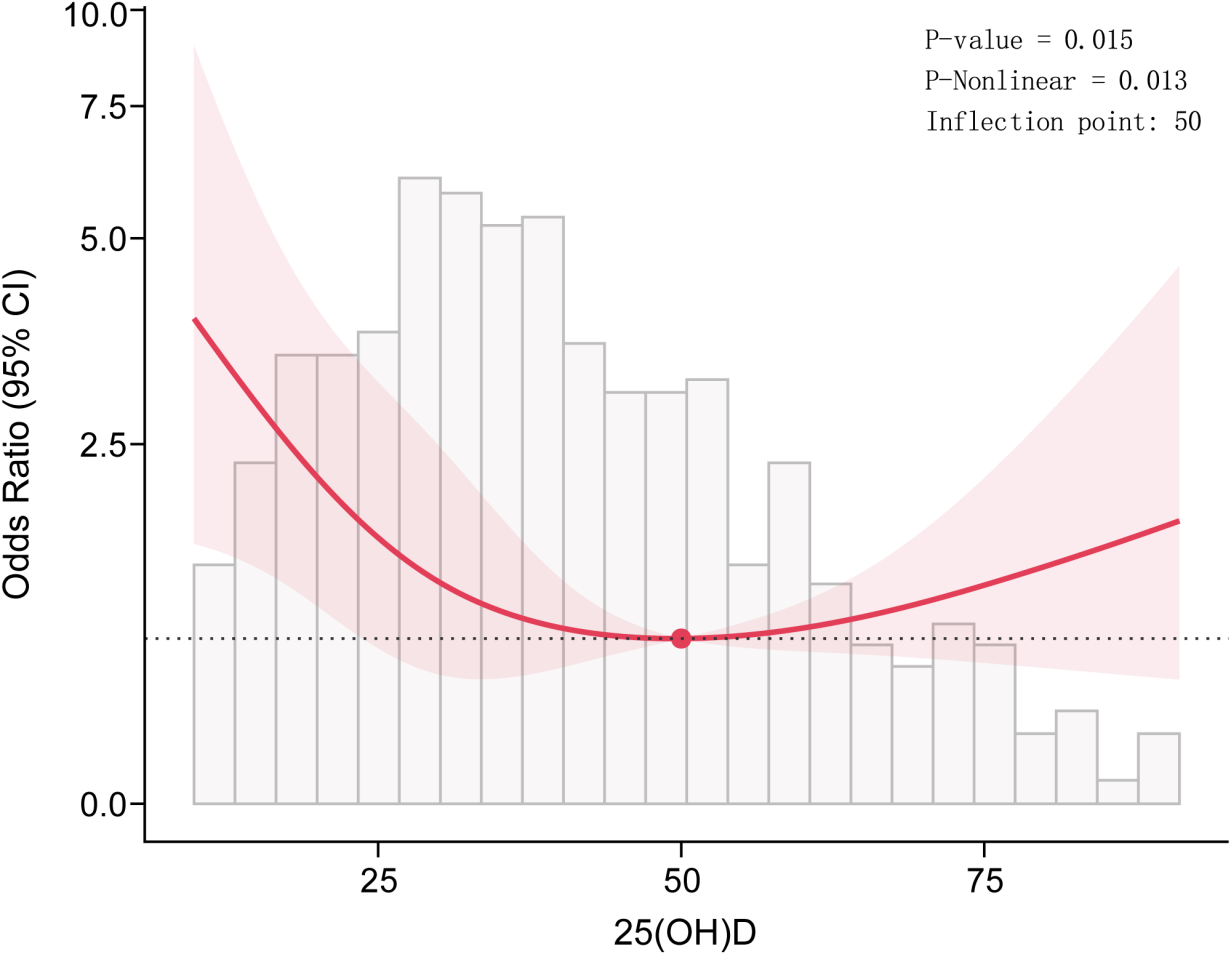
Restricted cubic spline curve depicting the association between 25(OH)D and cardiac autonomic dysfunction in DF. The analysis was adjusted for sex, age, diabetic duration, smoking history, BMI, MBP, HbA1c, TC, TG, eGFR, DN, DR, DPN, PAD, CVD, CAD, and HBP. 25(OH)D: 25‐hydroxyvitamin D; BMI: body mass index; CAD: coronary artery disease; CI: confidence interval; CVD: cerebrovascular disease; DF: diabetic foot; DN: diabetic nephropathy; DPN: diabetic peripheral neuropathy; DR: diabetic retinopathy; HbA1c: glycated hemoglobin; HBP: hypertension; MBP: mean blood pressure; PAD: peripheral arterial disease; TC: total cholesterol; TG: triglycerides.

To further investigate the relationship between 25(OH)D levels and cardiac autonomic dysfunction, we conducted a RCS regression analysis. The results of the RCS showed a “U” shaped relationship (*p* for nonlinear = 0.013) (Figure 2). When 25(OH)D levels were > 50 nmol/L, the odds ratio (OR) changed slowly, whereas it increased sharply when 25(OH)D levels were < 50 nmol/L. Moreover, segmented regression analysis was then performed for each group separately, with the results presented in Table 5. When 25(OH)D levels were < 50 nmol/L, the OR for predicted cardiac autonomic dysfunction per SD increase in 25(OH)D was 0.56 (95% CI: 0.44–0.72), while no significant effect was observed when 25(OH)D levels were > 50 nmol/L (Table 6).

**TABLE 5:** Effect of standardized 25(OH)D on cardiac autonomic dysfunction: adjusted ORs from segmented logistic regression analysis.

	OR per SD (95% CI)	*p*
25(OH)D < 50	0.56 (0.44–0.72)	< 0.001
25(OH)D ≥ 50	1.01 (0.67–1.54)	0.94

*Note:* ORs were adjusted for sex, age, diabetic duration, smoking history, BMI, MBP, HbA1c, TC, TG, eGFR, DN, DR, DPN, PAD, CVD, CAD, and HBP.

Abbreviations: 25(OH)D: 25‐hydroxyvitamin D; BMI: body mass index; CAD: coronary artery disease; CI: confidence interval; CVD: cerebrovascular disease; DN: diabetic nephropathy; DPN: diabetic peripheral neuropathy; DR: diabetic retinopathy; HbA1c: glycated hemoglobin; HBP: hypertension; MBP: mean blood pressure; OR: odds ratio; PAD: peripheral arterial disease; TC: total cholesterol; TG: triglycerides.

**TABLE 6:** Key take‐home messages for physicians.

Individuals with diabetic foot (DF) are particularly vulnerable to both vitamin D deficiency and cardiac autonomic dysfunction.
Lower 25‐hydroxyvitamin D (25(OH)D) levels are associated with impaired heart rate variability (HRV), including reduced SDNN, SDANN, LF, HF, and LF/HF ratio, in individuals with DF.
The association between 25(OH)D and cardiac autonomic dysfunction in individuals with DF is nonlinear, with a significantly increased risk when 25(OH)D levels < 50 nmol/L.
Targeted interventions for vitamin D deficiency in individuals with DF may be an important strategy to optimize cardiovascular risk management.

Abbreviations: 25(OH)D: 25‐hydroxyvitamin D; DF: diabetic foot; HF: high‐frequency power; HRV: heart rate variability; LF: low‐frequency power; LF/HF: rate of low‐frequency power between high‐frequency power; SDANN: the standard deviation of the 5‐min average RR intervals; SDNN: the standard deviation of normal sinus interval.

## 4 Discussion

The results of the current study demonstrate that lower 25(OH)D levels were associated with reduced HRV in individuals with DF. Specifically, compared to those with normal vitamin D levels, vitamin D deficiency was significantly associated with lower HRV indices, such as SDNN, SDANN, LF, and the LF/HF ratio. From normal vitamin D levels to deficiency, the abovementioned HRV indices decreased progressively. Interestingly, a nonlinear association between 25(OH)D levels and cardiac autonomic dysfunction was observed. A lower 25(OH)D level was identified as a risk factor for cardiac autonomic dysfunction when levels were < 50 nmol/L, whereas further decreases in 25(OH)D did not significantly increase the risk of cardiac autonomic dysfunction when levels were > 50 nmol/L. To the best of our knowledge, this is the first study that evaluates the association between cardiac autonomic dysfunction and vitamin D status in people with DF using HRV indices, which also provides a potential target for clinical interventions in this high‐risk population.

We found that people with DF at vitamin D deficient or insufficient status had a reduction in all time‐ and frequency‐domain HRV indices, except RMSSD and PNN50, compared to those with normal vitamin D levels. After adjusting for cardiovascular risk factors, a positive association between vitamin D levels and SDNN, SDANN, LF, and the LF/HF ratio persisted, although the association with HF was only borderline significant. Additionally, we found a graded decline in cardiovascular autonomic function as vitamin D status worsened, as evidenced by linear trends indicating that lower vitamin D levels were associated with lower HRV (e.g., SDNN, SDANN, LF, and LF/HF).

HRV reflects the interaction of the sympathetic and parasympathetic parts of the autonomic nervous system on the sinus node. Low HRV, a validated marker of cardiac autonomic dysfunction, is strongly associated with an increased risk of ventricular arrhythmias and sudden cardiac arrest [29, 30]. SDNN captures total HRV, reflecting both parasympathetic and sympathetic activity, while the LF/HF ratio serves as a valuable measure of sympathovagal balance. SDANN and LF power are measures of sympathetic activity, whereas RMSSD and HF power are measures of parasympathetic activity [23]. There is very limited data on the relationship between vitamin D status and cardiac autonomic function in people with DF. Until now, only a few studies have explored the relationship between vitamin D deficiency and HRV or CAN in apparently healthy populations or in high‐risk cardiovascular disease populations such as those with chronic renal failure or diabetes. Tak et al. analyzed serum 25(OH)D and HRV indices in 176 healthy subjects and found that 25(OH)D was positively correlated to SDNN, LF, and LF/HF, but not significantly correlated with HF. This is consistent with the findings of the present study [10]. Similarly, Chan‐Hee Jung et al. reported a positive correlation between serum 25(OH)D levels and SDNN in people with diabetes, suggesting that low vitamin D levels are related to an overall autonomic nervous system imbalance in diabetes [9]. However, they also showed RMSSD was significantly lower in people with vitamin D deficiency, which suggests main effects on the parasympathetic nervous system. In contrast, we found no significant association between 25(OH)D levels and RMSSD. Interestingly, Hansen et al. found an inverse U‐shaped association between 25(OH)D levels and HRV indices (e.g., SDNN, HF, RMSSD), indicating that both high and low levels of vitamin D could play a role in cardiac autonomic function [8]. Previous studies have reported age, retinopathy, nephropathy, HbA1c, BMI, TG, smoking, hypertension, and polyneuropathy as predictors of impaired cardiac autonomic function in people with diabetes [31, 32, 33]. Our study similarly found that poor glycemic control and micro‐ or macrovascular complications, including DN, DR, and PAD, may contribute to autonomic dysfunction in people with DF. These variations may stem from differences in study populations, methodologies, or confounding factors such as cardiovascular comorbidities and medication use. While previous studies primarily focused on healthy individuals or general diabetes populations, our study uniquely emphasizes people with DF, who may have a higher risk due to the progression of microvascular, macrovascular, and neuropathic complications, as well as a persistent chronic inflammatory state.

SDNN < 50 ms has been associated with increased mortality risk and adverse cardiovascular outcomes. Kleiger et al. demonstrated that SDNN < 50 ms predicted an approximately threefold increase in relative risk of all‐cause mortality at 2–4 years of follow‐up [24]. In the UK‐Heart study, among patients with chronic heart failure, those with an SDNN < 50 ms had an annual mortality rate of 51.4%, compared to only 5.5% for those with an SDNN > 100 ms [30]. In our study, SDNN < 50 ms was defined as cardiac autonomic dysfunction. Importantly, the incidence of cardiac autonomic dysfunction significantly increased when the 25(OH)D concentration was less than 50 nmol/L, which appears to be the turning point. Therefore, attention should be given to 25(OH)D levels in people with DF, and early intervention with supplementation is recommended, especially in those with vitamin D insufficiency or deficiency (which is currently defined as a 25(OH)D concentration less than 50 nmol/L). However, further large‐scale randomized controlled trials are needed to explore optimal 25(OH)D levels for the prevention of cardiac autonomic dysfunction, which is crucial for public health.

Although low serum 25(OH)D levels have been associated with an increased risk of cardiovascular disease and events, including ischemic cardiac events, cardiomyopathy, congestive heart failure, stroke, and even cardiovascular mortality in some studies, there is no clear evidence that vitamin D supplementation improves cardiovascular outcomes. Some randomized clinical trials showed that major cardiovascular events were not influenced by vitamin D supplementation, which consistently produced concordant results despite variations in target groups, baseline vitamin D status, and dosage regimens [34, 35, 36]. However, the potential impact of vitamin D supplementation on outcomes in people with DF, a population at high risk of cardiovascular mortality, has yet to be systematically investigated.

People with DF are at high risk for both vitamin D deficiency and cardiac autonomic dysfunction. Our findings, which demonstrate that low vitamin D levels in people with DF significantly increase the risk of cardiac autonomic dysfunction, are consistent with this association. This relationship may be explained through several interconnected mechanisms. Biologic effects of vitamin D mainly stem from its binding to the vitamin D receptor (VDR), present in nearly all tissues [37]. Vitamin D deficiency is known to contribute to chronic inflammation, endothelial dysfunction, and oxidative stress, such as decreasing inflammatory cytokines (tumor necrosis factor‐alpha and interleukin‐6) and oxidative stress markers (free radicals, nitric oxide), all of which play critical roles in the progression of both DF complications and autonomic neuropathy [38, 39, 40]. Additionally, vitamin D stimulates the production of nerve growth factor and enhances the function of calcium channels, which are important for neurotransmission and neuroprotection [41, 42]. Vitamin D may also increase insulin secretion and sensitivity and improve β‐cell function [43]. Moreover, evidence suggests that vitamin D deficiency activates the renin–angiotensin–aldosterone system, leading to increased vascular tone and arterial stiffness, both of which are strong predictors of overall cardiovascular disease risk [44]. In individuals with DF, the combination of chronic systemic inflammation, microvascular impairment, and prolonged metabolic dysregulation may be exacerbated by vitamin D deficiency, further increasing the susceptibility to cardiac autonomic dysfunction. These findings suggest that addressing vitamin D deficiency in people with DF may have potential benefits in reducing the risk of autonomic dysfunction, warranting further investigation.

This study has several notable strengths. First, it focuses on a high‐risk population, people with DF, who are particularly vulnerable to both vitamin D deficiency and autonomic dysfunction. By targeting this subgroup, the study provides valuable insights into a population that is often underrepresented in research but faces significant morbidity and mortality risks. Second, the use of RCS analysis allowed for the exploration of nonlinear relationships between vitamin D levels and HRV. This approach revealed a statistically significant threshold effect at vitamin D levels below 50 nmol/L. This finding provides a potential target for clinical interventions. Additionally, we adjusted for several key covariates, including age, diabetes duration, glycemic control, and cardiovascular risk factors, which are known to influence both vitamin D metabolism and autonomic function, minimizing potential confounding.

However, our study has several potential limitations. First, due to the cross‐sectional design of this study, causality cannot be determined. Longitudinal studies or RCTs are needed to determine whether vitamin D supplementation can improve HRV and reduce the risk of cardiac autonomic dysfunction in people with DF. Second, we assessed cardiac autonomic function using HRV, which was influenced by various factors such as physical fitness and medications. The interpretation of LF as a marker of sympathetic modulation remains controversial. Third, although we adjusted for key cardiovascular risk factors, unmeasured variables like physical activity, diet, and inflammation may still confound the results. These factors may influence both vitamin D status and HRV, particularly in individuals with DF who have limited mobility, potentially affecting the observed associations. However, we conducted 24‐h ECG Holter monitoring in individuals with DF during the proliferative phase of granulation tissue, when the inflammatory response is mild, potentially minimizing its impact on HRV. Lastly, while RCS analysis identified a significant threshold effect below 50 nmol/L, the relatively small sample size in this range may limit the robustness of the nonlinear association. Larger studies are needed to validate this threshold.

## 5 Conclusion

In conclusion, we found that lower 25(OH)D levels were associated with reduced HRV in people with DF. Specifically, when 25(OH)D levels are below 50 nmol/L, the risk of cardiac autonomic dysfunction in people with DF significantly increases as 25(OH)D levels decline. This highlights the threshold association and the potential of targeting 25(OH)D levels for clinical interventions in people with DF, a high‐risk group. However, further large‐scale studies are needed to confirm these findings and explore the efficacy of vitamin D supplementation in managing diabetic autonomic neuropathy.

## Author Contributions

Mingxin Bai performed statistical analyses and wrote the manuscript. Donge Yan, Ruixue Feng, Yan Liu, Murong Wu, Zhiyi Lei, and Sen He helped in statistical analysis and data collection and critically reviewed the manuscript. Xingwu Ran, Dawei Chen, Chun Wang, Lihong Chen, and Shuang Lin critically reviewed and edited the manuscript. Mingxin Bai, Yun Gao, and Donge Yan conceptualized the study concept and design and edited the manuscript. All authors have read and approved the final manuscript.

## Ethics Statement

The study was reviewed and approved by the Institutional Ethics Committee of West China Hospital and has been registered in the Clinical Trial Registry (registration number: CHiCTR2300076628). All patients provided written informed consent.

## Conflicts of Interest

The authors declare no conflicts of interest.

## Supporting information


**Table S1.** Associations between 25(OH)D and SDNN in individuals with diabetic foot.

## Data Availability

The datasets analyzed during the current study are available from the corresponding author on reasonable request.
