# Supplementary material for: Nonlinear Association Between Serum 25‐Hydroxyvitamin D and Cardiac Autonomic Dysfunction in Diabetic Foot: A Threshold Effect on Heart Rate Variability
Source: J Diabetes. 2025 Jun 10;17(6):e70109. doi: 10.1111/1753-0407.70109 (PMC12150134; doi:10.1111/1753-0407.70109)
Supplement: Supplementary file 1 — Table S1. Associations between 25(OH)D and SDNN in individuals with diabetic foot. [file JDB-17-e70109-s001.docx]

Supplementary Table Associations between 25(OH)D and SDNN in individuals with diabetic foot

|  | β, 95%CI | *P value* |
| --- | --- | --- |
| Sex, men | -0.019 (-0.063, 0.026) | 0.407 |
| Age | 0.001 (-0.001, 0.003) | 0.203 |
| Diabetic duration | 0.001 (-0.002, 0.003) | 0.581 |
| Smoking, yes | 0.002 (-0.039, 0.044) | 0.921 |
| BMI | 0.006 (0.000, 0.011) | 0.037 |
| MBP | -0.001 (-0.002, 0.000) | 0.204 |
| HbA1c | -0.015 (-0.022, -0.007) | <0.001 |
| TC | 0.009 (-0.004, 0.023) | 0.185 |
| TG | 0.007 (-0.008, 0.023) | 0.363 |
| eGFR | 0.000 (-0.001, 0.000) | 0.320 |
| DR | -0.052 (-0.085, -0.019) | 0.002 |
| DN | -0.058 (-0.094, -0.023) | 0.001 |
| DPN | -0.078 (-0.171, 0.014) | 0.098 |
| PAD | -0.070 (-0.105, -0.034) | <0.001 |
| CVD | -0.007 (-0.066, 0.052) | 0.807 |
| CAD | -0.015 (-0.059, 0.028) | 0.485 |
| HBP | 0.002 (-0.036, 0.040) | <0.902 |
| 25(OH)D | 0.001 (0.001, 0.002) | <0.001 |

BMI: body mass index; MBP: mean blood pressure; HbA1c: glycated hemoglobin; TC: total cholesterol; TG: triglycerides; eGFR: estimated glomerular filtration rate; DN: diabetic nephropathy; DR: diabetic retinopathy; DPN: diabetic peripheral neuropathy; PAD: peripheral arterial disease; CVD: cerebrovascular disease; CAD: coronary artery disease; HBP: hypertension; 25(OH)D: 25-hydroxyvitamin D;.
